# Iron Oxide Nanoparticles-Plant Insignia Synthesis with Favorable Biomedical Activities and Less Toxicity, in the “Era of the-Green”: A Systematic Review

**DOI:** 10.3390/pharmaceutics14040844

**Published:** 2022-04-12

**Authors:** Nadia M. Hamdy, Amira A. Boseila, Ahmed Ramadan, Emad B. Basalious

**Affiliations:** 1 Department of Biochemistry and Molecular Biology, Faculty of Pharmacy, Ain Shams University, Abassia, Cairo 11566, Egypt; 2 Department of Pharmaceutics, National Organization for Drug Control and Research (NODCAR), Cairo 12611, Egypt; amira.hamada@su.edu.eg; 3 Department of Pharmaceutics and Industrial Pharmacy, Faculty of Pharmacy, Sinai University, Sinai 41636, Egypt; 4 Department of Pharmaceutics and Industrial Pharmacy, Faculty of Pharmacy, Cairo University, Al Kasr El-Aini, Cairo 11562, Egypt; aamadan@pg.cu.edu.eg; 5 Mounir Armanious Research Center by EVA Pharma, Giza 12566, Egypt

**Keywords:** nanotechnology, plant-based green synthesis, iron oxide nanoparticles, toxicity, cancer hallmarks, antimicrobial, bioactivities

## Abstract

In the era of favoring environment-friendly approaches for pharmaceutical synthesis, “*green synthesis*” is expanding. Green-based nanomedicine (NM), being less toxic and if having biomedical acceptable activities, thence, the chemical methods of synthesis are to be replaced by plants for reductive synthesis. Iron oxide nanoparticles (IONPs) exhibited remarkable anti-microbial and anti-cancer properties, besides being a drug delivery tool. However, owing to limitations related to the chemical synthetic method, *plant-mediated green synthesis* has been recognized as a promising alternative synthetic method. This systematic review (SR) is addressing plant-based IONPs green synthesis, characteristics, and toxicity studies as well as their potential biomedical applications. Furthermore, the plant-based green-synthesized IONPs in comparison to nanoparticles (NPs) synthesized via other conventional methods, characteristics, and efficacy or toxicity profiles would be mentioned (if available). Search strategy design utilized electronic databases including Science Direct, PubMed, and Google Scholar search. Selection criteria included recent clinical studies, available in the English language, published till PROSPERO registration. After screening articles obtained by first electronic database search, by title, abstract and applying the PICO criteria, the search results yielded a total of 453 articles. After further full text filtrations only 48 articles were included. In conclusion, the current SR emphasizes the perspective of the IONPs plant-mediated green synthesis advantage(s) when utilized in the biomedical pharmaceutical field, with less toxicity.

## 1. Introduction

Recently, IONPs have provided various applications in the medical field [1]. Owing to the limitations of IONPs conventional synthetic methods, namely, methods of synthesis and toxicity, there has been a great emphasis on finding alternative methods for IONPs’ preparation. Literature has already reported on NPs’ drawbacks processed with either the attrition or pyrolysis conventional methods [2]. These drawbacks include the formation of defective surface structures, use of toxic chemicals, upon processing, damage to the environment and humans, low-rate production, and high cost. Plant-mediated IONPs synthesis emerged as a suitable alternative synthetic method, during the last decade.

**Review aim and objectives.** In an attempt to highlight the possible benefits of utilizing plant-mediated IONPs green synthesis, the review will compare NPs characteristics, efficacy, and the safety profile(s) of plant-mediated synthesis of IONPs to those prepared with the conventional methods.

The current interest in this SR is, first, to list different plant-mediated IONPs; second, to present their physical and chemical characteristics; third, the review will comprehend the most recent reported biomedical applications of plant-based green-synthesized IONPs. Furthermore, if applicable, toxicity studies conducted on plant-based green-synthesized IONPs are to be reported.

## 2. Methods

**Figure 1:**
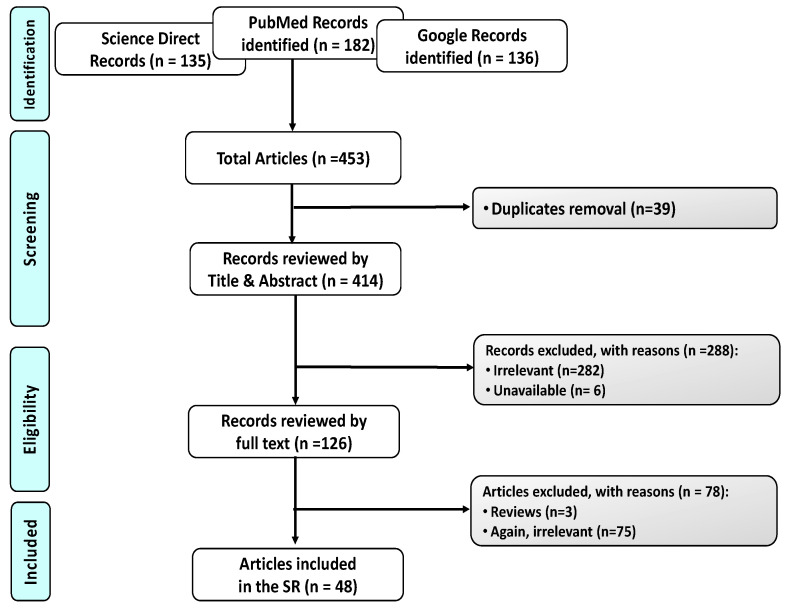
**PRISMA Flowchart** for the selection process, using three different databases for search namely, PubMed, Science Direct, and Google. A total of 453 articles were first identified. A total of 39 of them were duplicates; 288 excluded; 357 irrelevant to the current SR; 6 unavailable; 3 SR. Finally, 48 articles were eligible.

**Design.** Type of the Review. Intervention Systematic Review. This SR was performed in compliance with the preferred reporting items of SR PRISMA checklist (Figure 1). All steps were conducted in concordance with the Cochrane Handbook of SRs.

**Search strategy.** To identify all relevant interventional studies, but not SR studies, addressing “Green Plant-based IONPs Synthesis”.

**Data extraction.** Completed using the following keywords (“iron oxide”[Title/Abstract] OR “IONPs”[Title/Abstract] OR Fe_3_O_4_)[Title/Abstract] AND (nano[Title/Abstract] OR “NPs”)[Title/Abstract] AND (“green synthesis”[Title/Abstract] OR plant)[Title/Abstract]. Searched electronic databases were PubMed, ScienceDirect and Google Scholar. All records retrieved from e-database searches were downloaded locally and managed using the Mendeley X86 software facilities.

**SR Registration**. PROSPERO 2020 [CRD42020203760], obtained 11 September 2020.

**Inclusion criteria.** Studies that utilized plant-based synthesis for preparation of IONPs and studies that investigated the efficacy or toxicity of plant-based synthesis IONPs or investigated their clinical activity. **Exclusion criteria.** Studies that utilized only chemical processing for preparation of IONPs and other biogenic methods, not plant-based, were excluded.

**Studies selection.** Two independent authors (A.A.B. and A.R.) screened the literature search results for relevant studies according to the pre-specified inclusion and exclusion criteria. Any disagreement was resolved by the chief investigators (N.M.H. and E.B.B.). Any duplicates were removed using Mendeley X86 (Mendeley Desktop 1.19.8, by Mendeley Ltd. London, UK). Selected data were summarized using an Excel spreadsheet, full text articles or reports which do not meet the inclusion criteria were excluded and the reason(s) for exclusion are provided in the PRISMA flowchart (Figure 1).

**Data extraction/synthesis.** Data extracted included specific details about the context and the study method(s), with an emphasis on the current review specific objectives. Data synthesis was performed independently by (A.A.B. and A.R.) and, finally, checked by the chief investigators, to include all studies(s) that were found to be associated with green-synthesized plant-based IONPs and were summarized in Table 1, Table 2, Table 3, Table 4, Table 5 and Table 6.

## 3. Results

Figure 1 outlines the PRISMA Flowchart for the selection process.

Table 1 enumerates the different plants, whose extracts containing bioactive constituents ranging from essential oils, flavonoids, polysaccharides, alkaloids, terpenoids, minerals, amide glycosides, amino acids, fatty acids, sitosterol, tannins, glycosides, quinones, coumarins, carotenoid compounds, phenolic acid, lignans, saponins, and other polyphenols.

**Figure 2:**
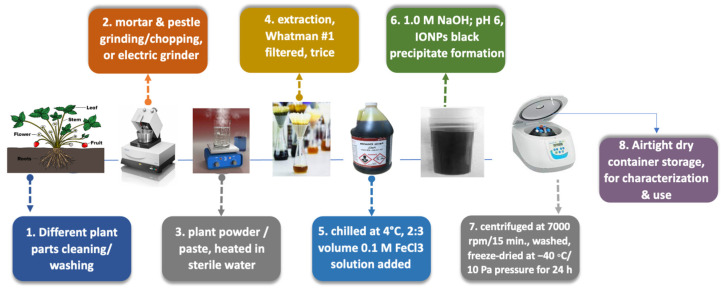
**Scheme for the mechanism of plant-based green synthesis IONPs.** Washing plants, drying, mortar and pestle grinding for chopping or an electric grinder, obtained powder or paste, heated in sterile water, complete extraction, Whatman no. 1 filtered, three times, chilled at 4 °C. A 2:3 volume ratio of 0.1 M FeCl_3_ solution is added, added 1.0 M NaOH for pH 6. IONPs black precipitate formation, centrifuged at 7000 rpm for 15 min., washed, freeze-dried at −40 °C at 10 Pa pressure for 24 h, airtight dry container storage, for further characterization and use.

Figure 2 illustrates the plant extract preparation and green IONPs synthesis, highlighting the effective chemicals in synthesis.

**Figure 3:**
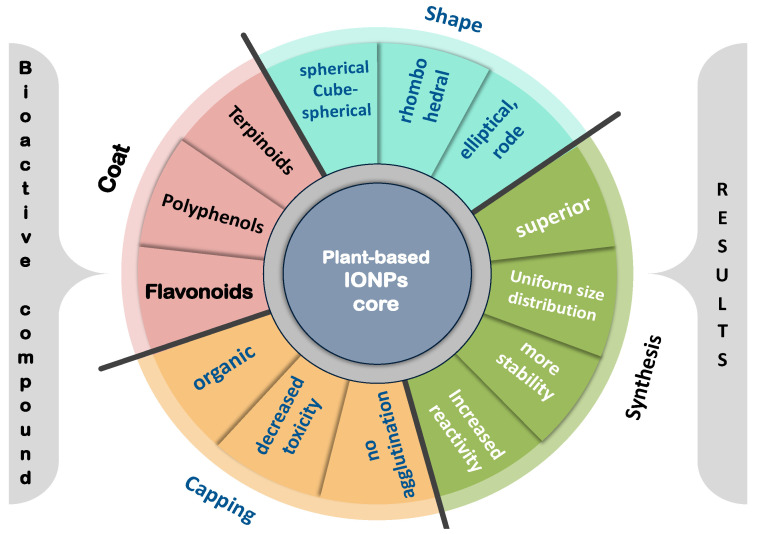
**Superiority of plant-based green-synthesized IONPs, regarding the coat, capping, shape and synthesis results.** Different important shapes of IONPs are either elliptical rode, cube-spherical vs. the chemical nature of the green coat used in NPs’ synthesis if flavonoids, polyphenols or terpenoids, which is the NP core, and capping effect with decreased toxicity and no agglutination, on the plant-based green-synthesized IONPs results or more stable particles with increased reactivity and uniform size distribution.

**Figure 4:**
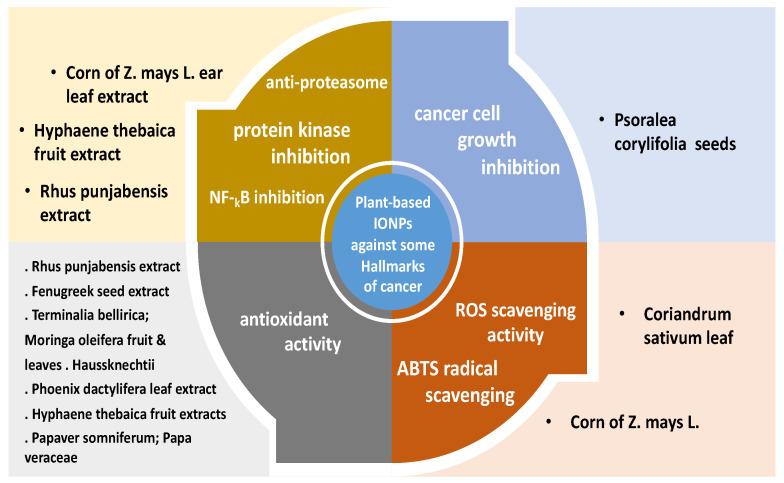
**Plant-based synthesized IONPs against some hallmarks of cancer.** Antioxidant effect, ROS scavenging activity, proteasome inhibition, protein kinase enzyme inhibition, NF-_k_B inhibition and cancer-cell growth inhibition are acted upon by plant-based green-synthesized IONPs, attributed to the activity of the IONPs as well as the biological plant compound(s) of corn of *Zea Mays* L., Fenugreek, Coriandrum leaf and Rhus extract, Papaver, Phoenix leaf extract, Hyphaene fruit extract, and finally Psoralea seeds.

Table 2, Table 3 and Table 4 summarize the different biomedical effects of green-synthesized IONPs, from different origins, stating the plant source, extract used, NPs’ particle size and shape, if available and also in Figure 3; antimicrobial effects in Table 2 and Table 3, or against some cancer hallmarks as presented in Table 4 and also in Figure 4.

Figure 3 enumerates the different important shapes of IONPs, the chemical nature of the green coat used in NPs’ synthesis (the NP core is our interest), and the capping effect on the plant-based green-synthesized IONPs.

The anti-bacterial effects of green-synthesized IONPs, from different plant origins, stating the plant source, extract used, NPs’ particle size and shape, if available, are summarized in Table 2.

Table 3 summarizes the anti-fungal effects of green-synthesized IONPs, from different plant origins, stating the plant source, extract used, NPs’ particle size and shape, if available.

The biomedical effects of plant-based green-synthesized IONPs against some cancer hallmarks are addressed in Table 4.

Figure 4 summarizes some cancer hallmarks acted upon by plant-based green-synthesized IONPs, attributed to the activity of the IONPs as well as the biological plant compound(s).

Table 5 lists articles tested plant-based green-synthesized IONPs in vivo and in vitro toxicities.

Six studies appeared in the SR current search obeying the inclusion criteria to be included in the review, where IONPs were plant-based green-synthesized, but without reported biomedical applications, as listed in Table 6.

## 4. Discussion

The usage of plant extracts, as a green method, for IONPs synthesis is environmentally favored as well as being a stable economic method for NPs synthesis [3]. To fully exploit the potential of green-NPs synthesis, a detailed understanding of the principles of green IONPs synthesis and high-throughput screening of stabilizing/capping agents on the physicochemical properties is required.

The superiority of plant extracts for NPs synthesis (Figure 2) is depicted in Figure 3. Plant extract advantage(s) are (1) to act as a capping or stabilizing agent, thus reducing the NPs size and improving their reactivity [4,5]. Moreover, (2) plant extracts contain bioactive constituents ranging from essential oils, flavonoids, polysaccharides, alkaloids, terpenoids, minerals, amide glycosides, amino acids, fatty acids, sitosterol, tannins, glycosides, quinones, coumarins, carotenoid compounds, phenolic acid, lignans, saponins, and other polyphenols (Table 1), which is a coat surface to the NPs core, preventing agglomeration, and thus, help obtain more uniform particle size distribution [2,4,6], keeping in mind the beneficial medically approved and recommended effects of these bio-active nutraceuticals, by themselves, potentiating the formed IONPs action, in comparison to chemical-based synthesis.

Plant parts used to prepare extracts could be the entire plant [1], but not limited to, pods [7], leaves [1,8,9,10,11,12,13,14,15,16,17], roots [18,19], seeds [20,21], fruits [22,23,24], fruit peel [22,43], berries [23], even seaweeds [25], as well as essential oils [26], honey [44], rhizomes [38], fulfilling the zero-waste concept, for more sustainability, as well as the fact that each part has its own beneficial effects and unique bioactive constituents.

Plant extract preparation, as illustrated in Figure 2, is executed after washing the plant part with tap water, then deionized water, to remove any dust or particulate matter. Second, after drying the plant parts well, they are chopped into small pieces [13], via mortar and pestle grinding [14,15,16,20] or homogenized by an electric grinder [7,8,9,24]. Finally, the obtained powder or paste is heated in sterile water for some time, till extraction is complete. The powder or paste is filtered through Whatman no. 1 filter paper, three times, and the obtained filtrate is chilled at 4 °C for further use.

Using plant extract to synthesize IONPs, through iron reduction and stabilization as NPs, by components of the plant extract, would give rise to either elliptical or spherical shapes, hexagonal [27], or cubical, rhomboidal shapes [28]. They could also exhibit a dendrimer shape, with branched surfaces at nanometer magnifications [29]; a structure may have resulted from chemical interactions such as hydrogen and electrostatic bonds between the organic capping agents of plant secondary metabolites and the IONPs core, a feature not present when IONPs were synthesized using the conventional methods.

The degree of plant-based green-synthesized IONPs aggregation is attributed to the super magnetic properties of iron, present with IONPs, competition between repulsive (electrostatic), and attractive (dipolar and Van der Waals) interactions among particles [7].

It is noteworthy mentioning that the IONPs plant-based green-synthesis relies on the co-precipitation method, in which iron ions, from iron chloride FeCl3, for example, are reduced in the presence of plant extracts. The plant extract might play the role of the reductant as well as the capping agent, due to the reducing properties of the bioactive constituents in the plant, minerals, and antioxidant properties as well.

However, the co-precipitation method has the physicochemical characteristics limitations, namely, poor crystallinity, low control on size, and polydispersity. However, the major advantage of using plant extract different moieties is in providing more biocompatible NPs to control the NPs diverse shapes.

The scheme in Figure 2 presents the mechanism of IONPs plant-based green synthesis. Where, a 2:3 volume ratio of 0.1 M FeCl_3_ solution is to be added to the leaf extract, and by adding 1.0 M NaOH to the solution, the pH changed from 3 to 6. The formation of IONPs indicated by the appearance of a black color precipitate, separated by centrifuging the solution at 7000 rpm for 15 min. The obtained black precipitate is washed and freeze-dried at −40 °C at 10 Pa pressure for 24 h. The as-obtained IONPs stored in an airtight dry container for further characterization and use.

Table 2 addresses plant-based green-synthesized IONPs biomedical efficacy studies with antimicrobial activity; anti-bacterial activity, where most of the current SR selected studies evaluated the anti-bacterial effect of green-synthesized IONPs, against Gram-positive and/or Gram-negative bacterial strains. Veeramanikandan et al. (2017) investigated the effect of plant-based green-synthesized IONPs using *Leucas aspera* leaf extract [16], where these IONPs strongly inhibited the Gram-positive bacteria; *Bacillus cereus*, *Staphylococcus aureus* and *Listeria monocytogens growth*. On the other hand, IONPs moderately inhibited the growth of Gram-negative bacteria; *Escherichia coli*, *Klebsiella pneumoniae*, *Proteus mirabilis*, *Salmonella enterica*, *Shigella flexneri*, *Vibrio cholera and Pseudomonas aeuroginosa*, at a concentration of 150 μg. However, IONPs showed a low inhibitory effect on the growth of *Bacillus cereus*. The antibacterial activity of IONPs synthesized using the aqueous fruit extracts of *Hyphaene thebaica*, is in the following order of *B. subtilis > P. aeruginosa > K. pneumonia > E. coli > S. epidermidis*, with an antimicrobial potential greater than the positive control (Erythromycin) [22].

The plant-based green-synthesized IONPs from the *Glycosmis Mauritania* leaf extract showed good anti-bacterial activity against *Bacillus cereus*, *B. subtilis*, *Enterococcus faecalis*, *Escherichia coli*, *Klebsiella pneumonia*, *Micrococcus luteus*, *Proteus mirabilis*, *P. vulgaris*, *Pseudomonas fluorescence*, *Staphylococcus aureus* and *Vibrio fluvialis* [8]. Being a popular source for green IONPs synthesis, the leaf aqueous extract of *Artemisia haussknechtii Boiss* showed a better effect against *E. coli* than *S. aureus* and *S. marcescens* [29]. The *Silda cordifolia* leaf extract mediated IONPs synthesis, and held potent antibacterial activity against various Gram-positive and Gram-negative bacteria; *E. coli*, *K. pneumoniae*, *B. subtilis*, and *S. aureus*, with comparable results to that of neomycin [12].

Lemon leaf extract possessed higher bactericidal activity compared to ampicillin, with greater anti-bacterial activity for *B. subtilis* (26.1 ± 0.24 mm inhibition zone) as compared to *K. pneumoniae* (21.5 ± 0.36 mm inhibition zone) [9].

*Couroupita guianensis* Aubl. fruit extract synthesized IONPs exhibited a greater anti-bacterial effect against Gram-negative *E. coli*, *S. typhi* and *K. penumoniae* than Gram-positive *S. aureus* [24]. *Ulva prolifera*-derived IONPs showed a better effect than chemo-IONPs against bacterial strains in the following order: *Staphylococcus epidermidis*, followed by *Bacillus subtilis*, and *Bacillus pumulis* [30]. Green-synthesized IONRs showed the maximum antibacterial activity for *S. aureus*, while the minimum for *P. aeruginosa* [23].

*Coriandrum sativum* leaf extract-synthesized IONPs showed good activity against *Micrococcus luteus* and *Staphylococcus aureus* [14].

*Psidium guavaja-Moringa oleifera* (PMS)-mediated IONPs inhibited *Staphylococcus aureus*, *Escherichia coli*, *Shigella*, *Pseudomonas aeruginosa*, *Salmonella typhi* and *Pasteurella multocida* growth [11].

Biogenic hematite NPs of average size <10 nm was synthesized using a green approach with *Aloe vera* extract (ALE). ALE-biogenic hematite NPs had prominent growth inhibition that is attributed to the smaller size, thinner protective layer surrounding the cells, higher dispersibility, and stability, owing to capping with organic moieties [28].

It is noteworthy to mention that the difference observed in the antibacterial potential of plant-based green-synthesized IONPs, against Gram-negative and Gram-positive bacteria, could be attributed to the differences in cell wall structure inherent in Gram-negative or Gram-positive bacteria. Where, Gram-positive bacterial cell wall possesses thick peptidoglycan layer, that contains teichoic and lipoteichoic acids, whereas, Gram-negative bacteria have thin peptidoglycan layer and the outer membrane contains lipopolysaccharides, phospholipids, and phosphoproteins.

Therefore, first, cannot conclude from the reported data if these NPs are more efficient against Gram-positive or against Gram-negative bacteria. Second, the antibacterial activity related to the plant-based synthesis cannot be ruled out. Third, the strength or specificity of the effect depends on the sensitivity of the microorganism. However, NPs, in particular oxide ones, show a tiny particle size and a large surface area, which can improve their antibacterial activity and surface reactivity, being a good choice nowadays following the prevalence of resistance to antibiotics. Therefore, a proposed recommendation for engineering IONPs with toxic surface modifiers, of plant origin or not, to improve their overall antimicrobial activity, is raised.

Plant-based green-synthesized IONPs with anti-fungal activity were reported in six articles. They checked the inhibitory effect of plant-based green-synthesized IONPs on fungi growth, as illustrated in Table 3, where these six studies mentioned the effect of green-synthesized IONPs on 11 different fungi. Plant-based green-synthesized IONPs exhibited anti-fungal activity via pronounced penetration ability through the fungal cell surface, followed by dissociation into respective ions, with generation of oxidative stress (OS) via production of reactive oxygen species (ROS), based on iron oxides within IONPs. Superoxide anions, hydroxyl radicals, and hydrogen peroxide (H_2_O_2_) are ROS, that can damage biological components such as DNA, proteins, and lipids. Moreover, iron oxides disrupt enzymatic reactions within the bacteria.

This was the case when using green-synthesized IONPs utilizing *Platanus orientalis* [39] against *Mucor piriformis* and *Aspergillus niger.*

It is worth mentioning that the current anti-fungal activity is based on the production of ROS and the overall generated OS, which is the opposite case, when using green-synthesized IONPs for cancer treatment.

IONPs that were green synthesized using *Laurus Nobilis* leaves reported inhibition zones of 13 and 14 mm against *Aspergillus Flavus* and *Penicillium spinulosumas*, respectively. Meanwhile, nystatin reported a 20-mm and 19-mm inhibition zones against them, respectively [27].

If better anti-fungal activity was observed, when using chemically synthesized IONPs in comparison to the green synthesized ones, this would be explained on the basis of smaller particle size.

As a perspective, it is worth mentioning that biologically synthesized IONPs with standard antibiotics (kanamycin and rifampicin) can exert synergistic effects against the five foodborne pathogenic bacteria, enabling a reduction in the dose of antibiotics, leading to decreased bacterial resistance and the overall mammalian cell toxicity [13]. Moreover, IONPs could be used in combination with conventional anti-fungal agents in the clinical setting, since the amount of both agents can be substantially reduced and thus, potentially avoid the adverse effects caused by the use of high doses of conventional anti-fungal drugs, with better efficiency. This is together with the retained plant metabolites activities as phenolic compounds generating more ROS to exert antimicrobial effect by themselves. Moreover, flavonoids hydroxyls are important for metal-binding activity.

Plant-based green-synthesized IONPs target some hallmarks of cancer, as enumerated in Table 3 and illustrated in Figure 4. IONPs synthesized using the corn (*Zea Mays* L.) displayed a dose-dependent anti-proteasome potential (large multi-catalytic proteinase complex located in the cytoplasm) [13]. Nuclear transcription factor kappa-B-cell (NF-_k_B) plays various roles in cellular homeostasis regulation. IONPs conventionally synthesized showed 50.3% NF-_k_B inhibition, while IONPs synthesized from *Rhus* plant extract showed 57.5% NF-_k_B inhibition [32]. Green-synthesized IONPs using the aqueous fruit extracts of *Hyphaene thebaica* were able to stop the activity of protein kinase enzyme [22]. The antioxidant activity (OS is one of the cancer hallmarks to be targeted) can be determined in vitro by measuring several free radical scavenging methods, such as 2,2′-azino-bis(3-ethyl benzo-thiazoline-6-sulphonic acid (ABTS), nitric oxide, 1,1-diphenyl-2-picrylhydrazyl (DPPH), as well as the reducing power assays [20,40]. Green biosynthesized magnetic IONPs using the corn (*Zea Mays* L.) ear leaves aqueous extract showed lower ABTS scavenging potential compared to standard [13]. When fenugreek seeds extract was used to prepare both silver and IONPs, silver NPs showed a higher antioxidant activity than IONPs syn. from fenugreek seeds extract [20].

When Alavi and Karimi compared the total antioxidant ability of IONPs, ascorbic acid, and the aqueous leaf extract of *A. haussknechtii*-synthesized IONPs, green-based IONPs exhibited the least antioxidant activity, followed by ascorbic acid, and the best was the leaf extract [29].

Therefore, we can say that plant-based IONPs synthesis showed moderate antioxidant activity in most of the studies in comparison with the plant aqueous extract, itself, used in their synthesis. This is due to the presence of different functional groups attached to IONPs surface, first, affecting its ability to react with ABTS radicals, second, due to the radicals’ stereo selectivity [13].

In addition, the small size of NPs and phytochemicals adsorbed on its surface (flavonoids, polyphenols, terpenoids, etc.), were responsible partially for the high, IONPs formed, antioxidant activity [20]. However, using ultrasonication for IONPs preparation resulted, in an even smaller particles’ size and thus, better antioxidant activity [14].

Plant-based green-synthesized IONPs in vitro toxicity studies listed in Table 4 showed that 4/48 studies investigated cytotoxicity on hepatocellular carcinoma HepG2 cell lines [7,10,24,42]. The spheroidal-shaped IONPs, with an average size of 17 nm, exhibited cytotoxic activity on HepG2 cell lines with IC50 of 44.51 µg/mL upon treatment for 24 h [24]. Meanwhile, Iqbal et al. reported an IC50 of 14.30 µg/mL with NPs size of 21 nm [10].

Three studies investigated cytotoxicity using HeLa cervical cancer cell lines [14,19,43]. Where Sathya et al. reported that the IONPs tested concentration to induce HeLa cervical cancer cell lines proliferation was IC50 of 155.3 µg/mL, Yusefi et al. reported that IONPs with a size below 11 nm were not reactive against HeLa cervical cancer cell lines, while Sharma et al. reported an IC50 of 41.9 µg/mL after 48 h incubation.

The cytotoxicity of green-synthesized IONPs was tested on WEHI164 fibro sarcoma cells, with no reported significant toxic effects up to the higher concentrations [44], which is a promising clue for their use in the biotechnology field, for drug delivery, and the other mentioned biomedical applications. The green-synthesized IONPs were not reactive to normal cell lines derived from human colon and kidney; CCD112 and HEK293, respectively, as well as to MCF7 for breast cancer, and the lung cancer cell lines (A549) [21,43]. Green-synthesized IONPs also showed no toxic effects towards macrophages upon incubation with IONPs different concentrations for longer periods of time up to 72 h [10,45].

One study evaluated the in vivo toxicity, with no significant changes in the biochemical parameters investigated, between the experimental and control groups [42]. Another study investigated activity for iron deficiency anemia [46]. Four studies have investigated the in vivo hemolytic activity of the green-synthesized IONPs and confirmed no hemolytic activity at lower concentrations [7,11,22,34].

It is worth mentioning that toxicity is related to either IONPs size or shape, where, the rod-shaped particles showed higher cytotoxicity when compared to the spherical-shaped particles. Six studies mainly presented synthesis, characterization, and reactivity of IONPs without testing their biomedical applications, as shown in Table 6.

**Limitation.** IONPs synthesized by magnetotactic bacteria, not included in the search, but are among the IONPs with the most remarkable physicochemical properties being superior from the point of view of crystallinity and magnetic properties to many IONPs obtained by chemical conventional methods. Second, the metrics of the green chemistry are not included, as the current review is a systematic rapid brief review, without meta-analysis as for Tobiszewski et al., 2014 (10.1039/C4AY00887A) and Tobiszewski et al., 2013 (10.1039/C3GC36976E).

## 5. Conclusions

Plant-based IONPs green synthesis is now evidenced to be relatively safer, sustainable, and of the least reported or no reported toxicity, when being compared to IONPs synthesized with the conventional chemical methods. Moreover, plant-based IONPs size and properties are superior to NPs synthesized by other methods. Together with the better chemical nature of the plant-based synthesis, IONPs are showing good reliable biomedical properties, namely, antimicrobial (antibacterial/anti-fungal) and anticancer effects.

Proteins, alkaloids, amino acids, alcoholic compounds, polyphenols (catechin, flavones, and phenolic acids), polysaccharides, organic acids, quinones, and other low molecular weight compounds, among sugars, terpenoids, polyphenols, alkaloids, phenolic acids, and proteins have all been implicated in the reduction of the iron ions into IONPs and in promoting their subsequent stability.

**Future prospective** is to address genetically engineered organisms for IONPs synthesis, with more biomedical applications.

## Figures and Tables

**Table 1:** List of the bioactive constituent(s) in different plants used for green synthesis of IONPs in the current SR.

Plant	Bioactive Constituent(s)	Ref.
*Carum carvi* L.	essential oils; monoterpenes, sesquiterpenes	[1]
Composite of *Psidium guavaja*; *Moringa oleifera*	flavonoids	[2]
Brown Seaweed, *Sargassum muticum*	polysaccharides	[3]
*Trigonella foenum-graecum*	saponin, alkaloids	[4]
*Kappaphycus alvarezii*	polysacharides	[5]
*Persea Americana*	polysacharides	[6]
*Papaver somniferum*; Papaveraceae	alkaloids	[7]
*Glycosmis mauritiana*; Rutaceae	Flavanone	[8]
Lemon; Citrus	triterpenoid	[9]
*Rhamnella gilgitica*	ω-phenylpentaene fatty acid amide diglycosides	[10]
Composite of *Psidium guavaja*; *Moringa oleifera*	phenolic acids, flavonoids, isothiocyanates, tannins, saponins	[11]
*Sida cordifolia*	alkaloids	[12]
corn of *Z. mays* L	flavonoid glycosides, polyphenols	[13]
*Coriandrum sativum*	terpenoids, polyphenols	[14]
*Coriandrum sativum*	flavonoids	[15]
*Leucas aspera*; Lamiaceae	alkaloids, flavonoids	[16]
Neem (*Azadirachta indica*)	sitosterol, polyphenolic flavonoids	[17]
*Skimmia laureola*	saponins, tanins, flavonoids	[18]
*Rheum emodi*	flavonoids	[19]
Fenugreek	saponins	[20]
Aloe vera/Flax seed	flavonoids, terpenoids, polysacharides, tannins, sterols/polysacharides, alkaloids	[21]
*Hyphaene thebaica*	phenols and flavonoids	[22]
Withania coagulans	alkaloids, steroidal compounds	[23]
*Couroupita guianensis*; Lecythidaceae	carotinoids and sterols	[24]
Green seaweeds, *U. prolifera*, *U. flexuosa*, *U. linza*, *U. intestinalis*, *U. clathrate*, one brown seaweed, *S. boveanum*	polysacharides	[25]
*Satureja hortensis*	essential oils; terpinene	[26]
*Laurus nobilis* L.	essential oils	[27]
Aloe vera	flavonoids, terpenoids, polysacharides, tannins, sterols/polysacharides, alkaloids	[28]
*A. haussknechtii*	essential oils; terpenes	[29]
Green seaweeds, *U. prolifera*, *U. flexuosa*, *U. linza*, *U. intestinalis*, *U. clathrate*, one brown seaweed, *S. boveanum*	polysacharides	[30]
*Terminalia bellirica*; Moringa oleifera	phenolic acids, flavonoids, isothiocyanates, tannins and saponins	[31]
*Rhus punjabensis*	polyphenols and flavonoid	[32]
*Lactobacillus casei*	flavons	[33]
*Punica granatum*	flavonoids, polysaccharides, tannins	[34]
*Lagenaria siceraria*	polyphenols	[35]
*Passiflora foetida*	flavonoids, alkaloids, glycosides and phenolic compounds	[36]
*Argemone mexicana*	flavonoids, oils	[37]
*Acorus calamus*	flavonoid, monoterpene, quinone, sesquiterpene, phenyl propanoid	[38]
*Platanus orientalis*; Platanaceae	flavonoids	[39]
*Phoenix dactylifera*	phenolic acids	[40]
*Psoralea corylifolia*	coumarins, flavonoids, meroterpenes	[41]
*Enterobacter sp. Mediated*	-	[42]
*Punica granatum*	flavonoids, complex polysaccharides, tannins	[43]
Honey	flavonoids	[44]
Coconut water	fatty acids, minerals and amino acids	[45]
-	-	[46]
*Musa ornate/Zea mays*	saponin, carotene, total flavonoid, lycopene, alkaloid and flavonoid/flavonoid glycosides, polyphenols	[47]
Green tea	lignans, minerals, polysacharides	[48]

**Table 2:** Plant-based green-synthesized IONPs with anti-bacterial activity.

Plant Family/Part Used	NPs Size/Morphology	Anti-Bacterial Effect against	Ref.
*Moringa oleifera (M. oleifera*)/ leaf and seed extracts	138–224 nm/Spherical	*Escherichia coli*	[2]
*Trigonella foenum-graecum*/ seed extract	11 nm/Spherical	Gram-negative *E. coli* and Gram-positive *S. aureus*	[4]
*Papaver somniferum*; Papaveraceae/-	38 ± 13 nm/Elliptical or Spherical	less activity against *Bacillus subtilis* and *Staphylococcus epidermidis*, *Klebsiella pneumonia and Pseudomonas aeruginosa*	[7]
*Glycosmis mauritiana*; Rutaceae/Leaves	≤100 nm = 58–79 nm/Spherical	*Bacillus cereus*, *B. subtilis*, *Enterococcus faecalis*, *Escherichia coli*, *Klebsiella pneumonia*, *Micrococcus luteus*, *Proteus mirabilis*, *P. vulgaris*, *Pseudomonas fluorescence*, *Staph. aureus* and *Vibrio fluvialis*	[8]
Lemon; Citrus/leaves	15–80 nm/Spherical	*B. subtilis* (Gram-positive) as compared to *K. pneumoniae* (Gram-negative)	[9]
*Rhamnella gilgitica*/leaves extract	21 nm/-	*B. subtilis* and *E. coli* and least effective against *P. aeruginosa.*	[10]
Composite of *Psidium guavaja*; *Moringa oleifera*/leaf	40–90 nm/Crystallite	*E. coli*, *S. typhi*, *S. aureus Shigella*	[11]
*Sida cordifolia*/ methanolic extract	20 nm/Spherical nano clusters	*E. coli*, *K. pneumoniae*, *B. subtilis*, and *S. aureus.*	[12]
corn of *Z. mays* L	-/Spherical	applications of IONPs with antibiotics exert synergistic effect, enabling antibiotics dose reduction, hence, decreased resistant bacteria or mammalian cell toxicity	[13]
*Coriandrum sativum*/leaf	20–90 nm/Spherical	*Micrococcus luteus* and *Staphylococcus aureus*	[14]
*Leucas aspera*; Lamiaceae/Leaves	20 nm/ Irregular rhombic	Gram-negative; *Escherichia coli* and *Klebsiella pneumoniae*, *Proteus mirabilis*, *Salmonella enterica*, *Shigella flexneri*, *Vibrio cholera* and *Pseudomonas aeuroginosa.*	[16]
*Skimmia laureola*/leaf extract	34 ± 0.37 nm/-	*Ralstonia solanacearum* in vitro and in planta	[18]
*Rheum emodi*/Roots	~12 nm/Spherical	*Escherichia coli* (Gram-negative) & *Staphylococcus aureus* (Gram-positive)	[19]
Fenugreek/seed extract	38–20 nm/Spherical	ineffective against *S. aureus* and *E. coli*	[20]
Withania coagulans/Berries	15–20 nm/Nanorods	*S. aureus and P. Aeruginosa*	[23]
*Couroupita guianensis*; Lecythidaceae/fruit extract	17 nm/Spherical	Gram-negative *E. coli* MTCC2939, *S. typhi* MTCC3917 and *K. penumoniae* MTCC 530 than Gram-positive *S. aureus* MTCC 96	[24]
*Ulva flexuosa* (wulfen); *J.Agardh*/aqueous extract.	12.3 nm/Cubo-spherical	strong antibacterial activity	[25]
*Laurus nobilis* L./leaf extract	8.03 ± 8.99 nm/Crystalline, spherical-like	*Listeria monocytogenes* (Gram-positive)	[27]
*Aloe vera*/ leaf extract	8.26 nm/Cubical, Rhomboidal, Spherical	*Pseudomonas aeruginosa*	[28]
*A. haussknechtii*/aqueous leaf extract	120–130 nm/Dendrimer	showed a bacteriostatic property at low concentration	[29]
*Terminalia bellirica*; *Moringa oleifera*/fruit and leaves	21.32–45 nm/Spherical	*E. coli*, *S. aureus*, *B. subtilis*, *P. aeruginosa*	[31]
Green seaweeds, *U. prolifera*, *U. flexuosa*, *U. linza*, *U. intestinalis*, *U. clathrate*, one brown seaweed, *S. boveanum*	10.05 ± 1.2 nm/Cubo-spherical crystalline	Gram-positive bacteria, except for five relatively resistant bacterial strains: *P. aeruginosa*, *K. pneumoniae*, and to some extent, *E. faecalis.*	[30]
*Rhus punjabensis*/extract	41.5 ± 5 nm/Rhombohedral crystal	antileishmanial and antibacterial activity	[32]
*Lactobacillus casei*/cytoplasmic extract	15 nm/Spherical	*Escherichia coli* and *Staphylococcus aureus*	[33]
*Punica granatum*/peel extract	-/Amorphous particles	*Pseudomonas aeruginosa*	[34]
*Lagenaria siceraria*/leaves	30–100 nm/Cube	Gram-negative; *Escherichia coli*, Gram-positive; *Staphylococcus*	[35]
*Passiflora foetida*/aqueous extract	10 to 16 nm/Spherical	Gram-negative; *Klebsiella pneumonia* and *Pseudomonas aeruginosa* and Gram-positive; *Bacillus cereus*, *Staphylococcus aureus* and *E. coli*	[36]
*Argemone mexicana*/leaf extract	10–30 nm/Spherical	Gram-positive; *E. coli* MTCC 443, *P. mirabilis* MTCC 425 and Gram-negative *B. subtilis* MTCC 441	[37]
*Acorus calamus*/rhizome	20–30 nm/Spherical clusters	*P. aeruginosa*	[38]

**Table 3:** Plant-based green-synthesized IONPs with anti-fungal activity.

Plant Family/Part Used	NPs Size/Morphology	Anti-Fungal Effect against	Ref.
*Papaver somniferum*; Papaveraceae/-	38 ± 13 nm/Spherical	*Fusarium solani*, *Aspergillus flavus*, *Aspergillus fumigates*, *Aspergillus niger* and *Mucormycosis*	[7]
*Ulva flexuosa*, *J.Agardh*/aqueous extract	12.3 nm/Cubo-spherical	moderate anti-fungal activity	[25]
*Satureja hortensis*/essential oil	10 nm/-	*Candida Albicans*	[26]
*Laurus nobilis* L./leaves extract	8.03 ± 8.99 nm/Spherical	*Aspergillus flavus* and *Penicillium spinulosum*	[27]
Green seaweeds; *U. prolifera*, *U. flexuosa*, *U. linza*, *U. intestinalis*, *U. clathrate*, one brown seaweed, *S. boveanum*	10.05 ± 1.2 nm/Cubo-spherical crystalline	*Candida Albicans* and *Aspergillus niger*	[30]
*Platanus orientalis*; Platanaceae/leaves	30–40 nm/Spherical	*Aspergillus niger* and *Mucor piriformis*	[39]

**Table 4:** Plant-based green-synthesized IONPs with anti-cancer activity.

Effect	Plant Family/Part Used	NPs Size/Morphology	Mechanism of Anti-Cancer Effects	Ref.
**Enzyme inhibitory**	Corn of *Z. mays* L. ear/leaf extract	-/Spherical	Strong proteasome inhibitory potential	[13]
	*Hyphaene thebaica*/ fruit extract	10 nm/Quasi-spherical	Protein kinase inhibition	[22]
	*Rhus punjabensis* extract	41.5 ± 5 nm/Rhombohedral	NF-_k_B inhibition	[32]
	*Psoralea corylifolia*/seeds	39 nm/-	Strong cancer cell growth inhibition in a dose-dependent manner using MDCK and Caki-2 cells	[41]
**Biomedical Antioxidant**	Corn of *Z. mays* L.	-/Spherical	Moderate ABTS radical scavenging	[13]
	Coriandrum sativum/leaf	20–90 nm/Spherical	ROS scavenging activity	[14]
	*Phoenix dactylifera*/leaf extract	2–30 nm/-	Moderate antioxidant activity	[40]
	*Hyphaene thebaica*/fruit extract	10 nm/Quasi-spherical		[22]
	*Papaver somniferum*; Papaveraceae/-	38 ± 13 nm/Elliptical or Spherical		[7]
	*A. haussknechtii*/aqueous leaf extract	120–130 nm/Dendrimer	Less antioxidant activity than Ag, Cu and TiO2 NPs	[29]
	Fenugreek/seed extract	20–38 nm/Spherical	Significant antioxidant activity	[20]
	*Terminalia bellirica*; Moringa oleifera/fruit and leaves	21–32–45 nm/Spherical		[31]
	*Rhus punjabensis* extract	41.5 ± 5 nm/Rhombohedral		[32]

**Table 5:** Plant-based green-synthesized IONPs in vivo and in vitro toxicity studies.

Plant Family/Part Used	NPs Size/Morphology	Toxicity Study	Ref.
*Papaver somniferum*; Papaveraceae/-	38 ± 13 nm/Elliptical or Spherical	Green IONPs showed superior biocompatibility with human RBCs as compared to chemical	[7]
*Rhamnella gilgitica*/leaves extract	21 nm/-	IONPs at 200 mg/mL inhibited macrophages growth by ~31%, confirming the non-toxic behavior	[10]
*Psidium guavaja; Moringa oleifera*/Leaf	40–90 nm/Crystallite	No hemolytic activity	[11]
*Coriandrum sativum*/Leaves	20–60 nm/Spherical	Using HeLa and Vero cell line; green IONPs showed less toxicity than the chemical synthesized	[15]
*Rheum emodi*/Roots	~12 nm/Spherical	Nontoxic using cervical (HeLa) cancer cells	[19]
Aloe Vera or Flax seed/leaves	30–50 nm/Spherical	Nontoxic to MCF-7 cells	[21]
*Hyphaene thebaica*/aqueous fruit extracts	10 nm/Quasi-Spherical	Brine shrimp’s cytotoxicity, cytotoxicity on L20B cells cell lines and no hemolytic activity	[22]
*Couroupita guianensis*; Lecythidaceae/fruit extract	7–80 nm/Spherical	Using HepG2 cell line, CGFE and CGFe_3_O_4_NPs IC50 were 120 and 44.51 µg/mL for a 24 h, respectively	[24]
Green seaweeds, *U. prolifera*, *U. flexuosa*, *U. linza*, *U. intestinalis*, *U. clathrate*, one brown seaweed, *S. boveanum*	10.05 ± 1.2 nm/Cubo-spherical	No acute toxicity in Artemia and no toxic potential in barnacle, with considering the biocompatibility preference of bio-IONPs	[25]
*Rhus punjabensis* extract	41.5 ± 5 nm/Rhombohedral crystal	Lower cytotoxic effect against HL-60 leukemic and DU-145 prostate cancer cell lines	[32]
*Punica granatum*/peel extract	-/-	No hemolytic activity on RBCs of male albino rats	[34]
Enterobacter sp. Mediated Synthesis/-	1.4 nm/Spherical	Non-toxic to Hep-G2 cells or male Sprague-Dawley rats	[42]
*Punica Granatum*/fruit peel extract	Below 11 nm/-	Nontoxic using MCF7, HeLa and lung (A549) cancer cell lines and two normal cell lines CCD112 and HEK293	[43]
Honey	2.22–3.21 nm/Spherical	Using WEHI164 fibro sarcoma cells, no significant toxicity in higher concentration up to 140 ppm	[44]
Coconut water/-	3.8 nm/Spherical	No toxicity to macrophage cultures, conc. to 300 μg/mL	[45]
*Musa ornate* and *Zea mays*/ aqueous leaf extract	-/-	Using Vero, PK15 and MDBK cells, maximum cell viability was at 50 μg/100 μL IONPs, with no toxicity	[47]

**Table 6:** Studies addressing plant-based green-synthesized IONPs without biomedical application(s).

Plant Family/Part Used	NPs Size/Morphology	Ref.
*Carum carvi* L./entire plant	<300 nm/Spherical	[1]
Brown Seaweed, *Sargassum muticum*/entire seaweed	18 ± 4 nm/Crystalline cubic	[3]
*Kappaphycus alvarezii*/entire seaweed	14.7 nm/Spherical	[5]
*Persea Americana*/Seeds	-/Nanorods	[6]
Neem (*Azadirachta indica*)/Leaf	9–12 nm/Irregular	[17]
Green tea/leaf extract	84.7 ± 11.5 and 117.8 ± 26.2 nm/Spherical	[48]

## Data Availability

Not applicable.
